# Tertiary lymphoid structures in pancreatic cancer: a new target for immunotherapy

**DOI:** 10.3389/fimmu.2023.1222719

**Published:** 2023-07-17

**Authors:** Xinlei Zou, Canghai Guan, Jianjun Gao, Wujiang Shi, Yunfu Cui, Xiangyu Zhong

**Affiliations:** Department of Hepatopancreatobiary Surgery, The 2nd Affiliated Hospital of Harbin Medical University, Harbin, China

**Keywords:** tertiary lymphoid structure, pancreatic cancer, microenvironment, immunotherapy, prediction

## Abstract

Pancreatic cancer (PC) is extremely malignant and shows limited response to available immunotherapies due to the hypoxic and immunosuppressive nature of its tumor microenvironment (TME). The aggregation of immune cells (B cells, T cells, dendritic cells, etc.), which is induced in various chronic inflammatory settings such as infection, inflammation, and tumors, is known as the tertiary lymphoid structure (TLS). Several studies have shown that TLSs can be found in both intra- and peritumor tissues of PC. The role of TLSs in peritumor tissues in tumors remains unclear, though intratumoral TLSs are known to play an active role in a variety of tumors, including PC. The formation of intratumoral TLSs in PC is associated with a good prognosis. In addition, TLSs can be used as an indicator to assess the effectiveness of treatment. Targeted induction of TLS formation may become a new avenue of immunotherapy for PC. This review summarizes the formation, characteristics, relevant clinical outcomes, and clinical applications of TLSs in the pancreatic TME. We aim to provide new ideas for future immunotherapy of PC.

## 1 Introduction

The theory that tumor cells act as “seeds” capable of interacting with a specific microenvironment “soil” was first proposed by Stephen Paget in 1889 (1), and scientists have subsequently proposed the concept of the tumor microenvironment (TME). The TME is the environment in which tumor cells live and interact with each other. Several studies have shown that the TME contributes significantly to tumor invasion, metastasis, drug resistance, and immune escape (2–4). One of the key elements of the TME is immune cells, which mostly include T cells, B cells, natural killer cells (NK), and macrophages (5). As innate immune cells, NK cells and macrophages are often the first to kill and remove tumor cells and have antigen-presenting properties, further activating lymphocytes in adaptive immunity and promoting antitumor immune effects (6). T cells and B cells, as part of adaptive immune cells, contribute to the antitumor process (7). Tumor-infiltrating lymphocytes (TILs) are lymphocytes specific for autologous tumor cells with multiple phenotypes (CD4+ T, CD8+ T, Treg, and B cells) (8). Their number, type, and functional status may determine the susceptibility of tumors to the immune response. The tertiary lymphoid structure (TLS) consists of an orderly aggregation of T cells, B cells, dendritic cells (DCs), and other immune cells with high endothelial venule (HEV) structures. TLSs are also known as tertiary lymphoid organs (TLOs) or ectopic lymphoid structures (9). TLSs were identified in autoimmune diseases, particularly in the synovial tissue of patients with rheumatoid arthritis (10). The prognostic impact of TLSs is somewhat variable across diseases, being associated with good prognosis in a variety of tumors (11), such as hepatocellular carcinoma (12), melanoma (13), colorectal cancer (14), and breast cancer (15), while being able to promote progression of some immune-related diseases because related immune-enhancing effects are associated with poor prognosis (16).

Pancreatic cancer (PC) is one of the most aggressive tumors. Pancreatic ductal adenocarcinoma (PDAC) accounts for 90% of PC cases (17). The PC in this review refers specifically to the PDAC. According to the latest statistics from the American Cancer Society, the 5-year survival rate is 12% (18). Except for early resection as radical treatment, chemotherapy and radiotherapy have limited effects on the treatment of PC (19). The PC microenvironment is extremely complex and heterogeneous, with dense desmoplasia and a large number of immunosuppressive cells. Tumor cells can proliferate in their microenvironment and undergo immune escape (20). Immunotherapy is gradually becoming a new direction in the treatment of PC (21). However, due to the characteristics of its TME, therapeutic strategies are not effective for all PC patients, and the development of drug resistance and the lack of antitumor T-cell function and number are still problems with most immunotherapy modalities (22, 23). TLSs, as one of the hot topics in tumor immunity, are present in PC tissues and closely associated with the prognosis of PC patients. TLSs can even become a new target in immunotherapy for PC. Therefore, we summarize the formation process, clinical outcomes, and influencing factors to provide information for future TLS-related studies.

## 2 Pancreatic cancer and TILs

TILs mainly include T cells, B cells, and NK cells (24, 25). T and B cells comprise the bulk of the cells in TLSs. Therefore, this section mainly provides an overview of these two TIL subpopulations in the pancreatic TME.

### 2.1 T cells

Numerous studies have demonstrated that T cells are crucial to the antitumor immune response. Naive T cells are mainly divided into CD4+αβ and CD8+αβ cells. When encountering an antigen, naive CD8+ and CD4+ T cells differentiate into effector cells, which migrate to the site of infection or the tumor (26–28).

CD8+ T cells are also known as cytotoxic T lymphocytes (CTLs). CTLs clear tumor cells mainly through the Fas–FasL and perforin–granzyme pathways. In addition, IFN-γ, IL-2, IL-12, and TNF-α are involved in this process (29). More CD8+ T cells are associated with prolonged disease-free survival (DFS) and overall survival (OS). Their degree of infiltration is inversely related to the stage of the tumor. One study revealed that CD4+ T lymphocytes with potential T-helper capacity exist near CD8+ T cells (30). CD4+ T cells can be further differentiated into types with different functions: Th1, Th2, Th17, Th22, Treg, and Tfh cells (31). Th1 cells secrete IL-2, IL-10, TNF-α, and IFN-γ to participate in the antitumor immune process (32, 33). IFN-γ promotes CTL action as well as activation of M1 macrophages with antitumor effects (34). In contrast to Th1 cells, Th2 cells dominate the PDAC microenvironment, acting mainly through the cytokines IL-4, IL-5, and IL-13 (35). Involvement in the activation of M2 macrophages is associated with the immunosuppressive and fibrotic properties of the PC microenvironment (36, 37). Th17 cells have protumorigenic effects in various tumors, including PC (38, 39); they mostly act in the early stage of the tumor, with IL-17 and IL-22 as effector molecules of Th17. Among them, IL-17 promotes the stemness of PC cells by inducing DCLK1 and ALDH1A1 expression (40). Th22 cells are significantly increased in PC tissues, and their production of IL-22 is able to promote tumor development. High levels of Th22 and IL-22 are associated with PC lymph node metastasis, TNM stage, and poorer patient survival (41, 42). Increased CD4+CD25+FOXP3+ T cells (Tregs) in tumors as well as in peritumor tissues are associated with poor patient prognosis (43), and Tregs promote PC progression by upregulating immunosuppressive factors (IL-10 and TGF-β) and CTLA-4. They suppress the antitumor immunity of local CD8+ T cells in tumors (44). Tfh cells are able to exert antitumor effects and are associated with better prognosis in PC patients. They promote the antitumor humoral immune action of B cells by producing CXCL13 to recruit CD8+ T cells and B cells (45). It has been partially investigated whether Tfh cells are able to promote TLS formation in a variety of tumors. In ovarian cancer, promotion of Tfh cell differentiation induces larger TLS formation as well as B-cell recruitment, along with increased FAS and GL7 expression (46); in non-small cell lung cancer (NSCLC), the interaction between Tfh cells and B cells *via* the CXCL13-CXCR5 axis induces smaller TLS formation within tumors (47). A similar result was also found in a mouse model of colorectal cancer (48). In contrast, blocking CXCL13 impairs the recruitment of B cells induced by Tfh cells, and the effect of promoting TLS formation is abrogated (49). In addition, the ability of Tfh cells to produce IL-21, which promotes B-cell activation, supports the function of CTLs (**Table 1**). Immune checkpoint expression is a form of immunosuppression. Premature overexpression of PD-1/PDL-1, CTLA-4, LAG-3, and TIM-3 in tumors and T cells can contribute to tumor survival and propagation (50).

**Table 1:** The role of different phenotypes of T cells in antitumor therapy.

	T-cell phenotype	Main effector molecules	Antitumor effect
CTL	CD8+ T cells	Fas–FasL and perforin–granzyme pathway; IFN-γ, IL-2, IL-12, and TNF-α	Promotion
Helper T-cell	Th1 cells	IL-2, IL-10, TNF-α, and IFN-γ	Promotion
	Th2 cells	IL-4, IL-5, and IL-13	Inhibition
	Th17 cells	IL-17 and IL-22	Inhibition
	Th22 cells	IL-22	Inhibition
	Treg cells	IL-10, TGF-β, and CTLA-4	Inhibition
	Tfh cells	CXCL13 and IL-21	Promotion

### 2.2 B cells

Although CD20+ B cells in TLSs are related to positive patient prognosis, their function in the TME is not straightforward (51). B cells usually exhibit antitumor-related effects such as antigen presentation and antibody production in humans. However, the presence of B cells is often associated with poor prognosis in a variety of mouse tumor models. B cells promote M2 macrophage polarization through BTK signaling (52), and B cell-derived IL-35 contributes to tumor cell proliferation (53). CXC13 (B-cell chemotactic agent) production in the TME may be influenced by the hypoxic microenvironment, leading to CD20+ B-cell infiltration and promoting the development of PDAC (54). CD20+ B cells in the TME (CD20+ B cells in non-TLS) do not suggest a good prognosis for PC patients. Regulatory B cells (CD19+CD39+CD73+ Bregs), which have the highest frequency of CD39/CD73 expression among immune cells, are able to actively produce immunosuppressive adenosine, which decreases the antitumor immune response (55). Bregs act through the immunosuppressive factors TGF-β, IL-4, and IL-10 with other immune cells, causing immunosuppression-related polarization of T cells and macrophages and thus promoting tumor progression (56).

## 3 Pancreatic cancer and TLS

### 3.1 Identification of immune cell subpopulations and TLSs

Histological assessment of TILs in the PC microenvironment needs to take into account immune and inflammatory cell interactions in the TME (57). There has been limited progress in studies on assessing TILs in PC (58, 59). Currently, the most commonly used modality is the detection of different immune subpopulations by immunohistochemistry and immunofluorescence (IF) in tumor tissue samples. Antibodies are used to detect specific protein antigens in tissue samples to identify the major TIL subgroup classes, distribution, and localization in the tissue (60). Digital Pathology Image Analysis System analyzes tissue images stained for different immune populations, such as CD20, CD3, CD4, CD8, and FOXP3, to quickly and accurately measure the number and location of specific cell types (13, 61). Identification of two or more lymphoid aggregates that contain both CD20+ B cells and CD3+ T cells is required to define early-stage TLS (ES-TLS); mature TLSs (M-TLS) appear as germinal centers (GCs) and HEVs. Finally, by measuring the entire area of PDAC tissue as well as the area of TLOs at various locations (intratumoral and peritumoral), it is possible to compute the ratio of total TLSs to total PC tissue (62). Most current TLS assessment methods are based on image segmentation of H&E slices, a process that requires extensive training of images and computational power to obtain more accurate data (63). Although there are few studies on TLS assessment methods specific to PC, there are references to other tumor identification methods. For example, Silina et al. quantified TLSs at different stages by combining multiple parameters using Inform software (64). Panagiotis used a modified DeepLab v3+ network to analyze lung cancer tissue H&E images for detecting candidate TLS regions and was able to overfilter false-positive TLS regions, with high sensitivity and specificity (65).

### 3.2 Formation of TLSs

TLSs resemble secondary lymphoid organs (SLOs) in terms of structure, with B cells, T cells, DCs, macrophages, and structures such as GCs and HEVs (66). Unlike SLOs, TLSs do not have a membrane structure, which allows them direct contact with the site of inflammation (67). Immune cells in TLSs enter directly into the inflammatory environment, allowing for a rapid and intense immune response.

TLSs are formed under the regulation of various chemokines, cytokines, and adhesion molecules. In chronic inflammatory settings (autoimmune diseases, viral infections, tumors, etc.) (68–70), immune cells such as neutrophils, eosinophils, and monocytes accumulate at sites of inflammation. The ILC3 family is the major ILC population and is involved in the immune response *in vivo*; lymphoid tissue inducer (LTi) cells, as part of the ILC3 family, are ILC members essential for embryonic lymph node (LN) formation (71). CXCL13 plays an important role in the aggregation of LTi cells in most LN progenitors (except mesenteric LN); when lymphatic endothelial cells secrete the ligand CCL21, CCR7 is effective in promoting LTi cell aggregation (72). CXCL13 and IL-7 bind to CXCR5 and IL-7 receptors on the surface of the LTi to promote LTi aggregation and activation (73). Cellular inflammatory factors and lymphatic chemokines (CXCL13, CCL21, CCL19, and CXCL12) produced by LTi function together to coordinate the early recruitment of T cells and B cells to form early aggregates (74). follicular dendritic cells (FDCs) are derived from PDGFRβ+ cells in perivascular non-lymphoid organs, and signaling by LT and TNF family members can promote PDGFRβ+ preFDC maturation when exposed to the appropriate environment. LTi cells and B cells express most TNF ligands; LTi cells provide insufficient factors, but B cells may provide sufficient cytokines to promote end-stage FDC differentiation. With continued inflammation-mediated stimulation, followed by follicular dendritic cell (FDC) enrichment, isolated FDCs expressing CCL19 and/or CCL21 interaction with CCR7-expressing T cells and CXCL13-expressing FDCs interact with CXCR5-expressing B cells, allowing T cells and B cells to form compartments. GCs form during the maturation phase of TLSs (75). FDCs and B cells continuously present antigens to maintain TLSs (76). Macrophages and endothelial cells promote expression (VCAM-1 and ICAM-1) and secretion (IL-8, CCL2, and CCL20) of various substances and thus formation of TLSs in gastrointestinal tumors by secreting the cytokine IL-36γ (77). While CD68+ macrophages are associated with poor prognosis, a significant decrease in CD68+ macrophage infiltration has been found in hepatocellular carcinoma peritumoral TLS (78). High expression of CCL5 significantly suppresses the development of tumor cells, but its ability to increase the ratio of M2/M1 macrophages promotes the progression of cancer (79). DCs promote lymphocyte infiltration and production of TLSs, inhibit tumor growth, and play an antigen-presenting role in TLSs. In tumors with poor DC infiltration, TIL cell density is significantly reduced, with poor prognosis (80).

LTi plays a major role in the development of both TLS and SLO. LTi expresses LTα1β2 on its surface, which binds to LTβR expressed on stromal cells. Subsequently, it promotes stromal cell secretion of vascular endothelial growth factor C (VEGFC) to induce HEV formation (81, 82). However, some studies have indicated that VEGF blockade can promote HEV and TLS formation (83, 84). LTα1β2 expression in T cells is induced by CCL21 and CCL19; CXCL13 promotes the expression of LTα1β2 in B cells. These B cells then can enter TLSs by HEV *via* the LTβR signaling-dependent pathway (85). Vascular adhesion factors such as VCAM1 and MADCAM1 contribute to the release and vascularization of homeostatic chemokines from HEVs (77), which also contribute to lymphatic neogenesis as well as to the maintenance of TLSs (**Table 2**).

**Table 2:** Cytokines and chemokines involved in the formation of TLS.

Chemokines/Cytokines	Origination	Receptors/target cells	Function
CXCL13	Stromal cells	CXCR5	Promote LTi aggregation and activation; coordinate the early recruitment of T and B cells to form early aggregates
CCL21	Stromal cells	CCR7	
CCL19	Stromal cells	CCR7	
CXCL12	Stromal cells	–	
IL-7	–	IL-7R	Promote LTi aggregation and activation
TGF-β and IL-6	–	Th17 cells and Treg cells	Promote Th17 cell differentiation and inhibit Treg cell differentiation
IL-27	–	Th17 cells	Restrict the size and function of TLS
IL-17 and IL-22	Th17 cells	–	Production and development of TLS
IL-36γ	Macrophages	–	Promote formation of TLS

TLS, tertiary lymphoid structure.

**Figure 1:**
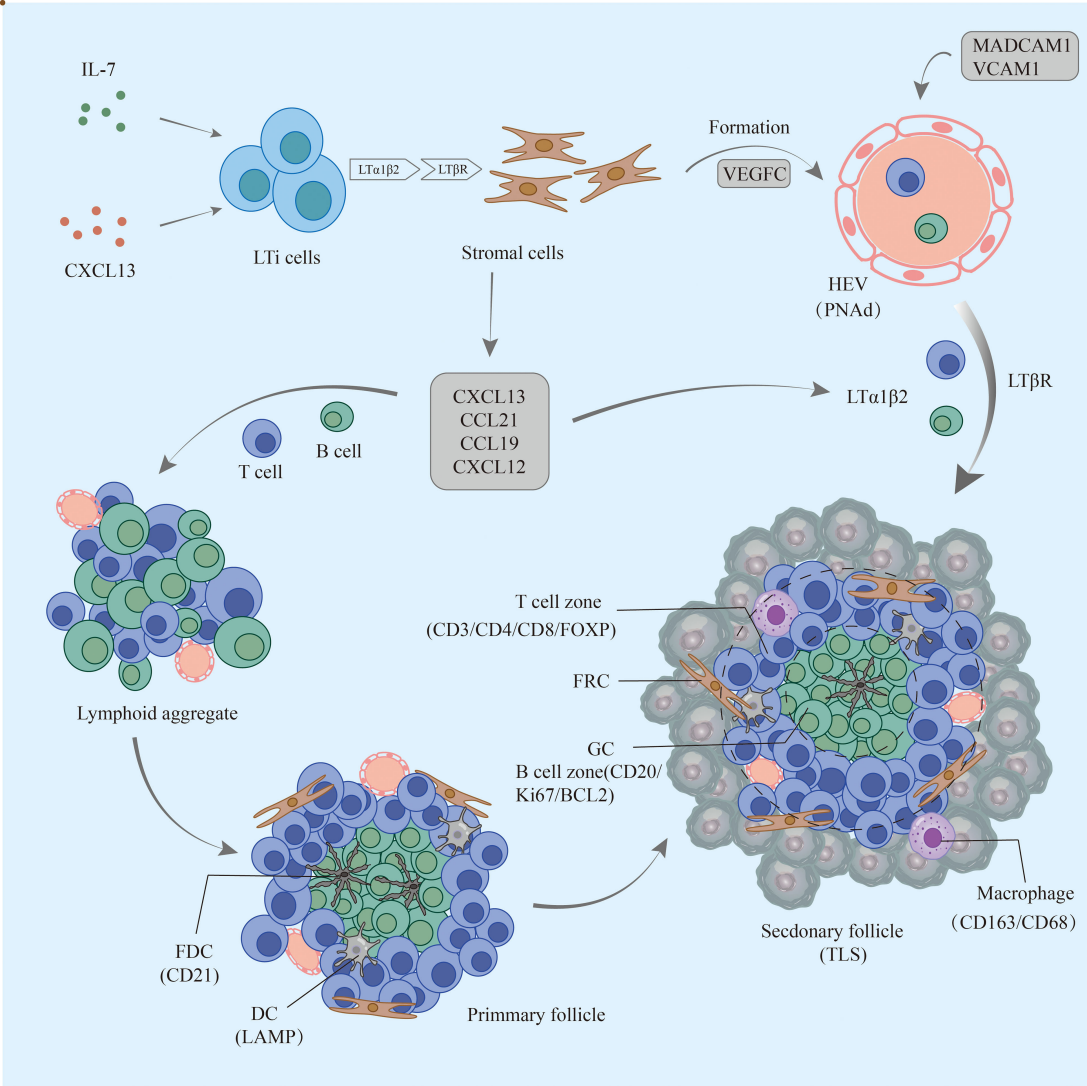
The formation process of TLSs in pancreatic cancer. Early lymphoid aggregates do not have FDCs and advanced structures. Under continuous stimulation by the tumor, FDCs are enriched and gradually form T-cell and B-cell compartments but not GCs; eventually, mature TLSs develop GCs, HEVs, and other structures and play a role in the antitumor process. TLSs, tertiary lymphoid structures; FDCs, follicular dendritic cells; GCs, germinal centers; HEVs, high endothelial venules.

Although LTi plays an important role, it is not indispensable in the formation of TLSs, and other factors also induce TLS formation (86). The combined effect of TGF-β and IL-6 is to promote Th17 differentiation and inhibit Treg differentiation (87, 88). Moreover, several studies have found that Treg deficiency promotes TLS formation and T-cell proliferation in tumors (89). In addition, IL-27 can restrict the size and function of TLSs by limiting the proliferation of Th17 cells (90). Th17 cells and the cytokines (IL-17 and IL-22) they secrete are also involved in the production and development of TLSs (91). IL-17 induces the expression of CXCL13 and CCL19 in mouse stromal cells in response to microbial stimulation, contributing to the formation of TLSs in lung tissue. In conclusion, there are potential inducers of TLS formation beyond LTi in allograft rejection, autoimmunity, chronic inflammation, or tumor settings (92) (**Figure 1**).

### 3.3 Features and functions of TLS

A growing number of studies point to TLSs as sites of plasma cell antigen presentation, T-cell activation, B-cell maturation, and differentiation (93). Some studies have found that TLSs with localization within a tumor are less common in PDAC but that peritumor TLSs are relatively common. Other studies have shown that TLSs are commonly found in PDAC tissue (94). Thus, the presence of TLSs is somewhat heterogeneous, with differences in cell composition, structure, and number in different tumors and patients.

ES-TLSs exhibit varying degrees of B-cell and T-cell aggregation but lack FDCs as well as higher-level structures (GC, HEVs, etc.). They can be present in various locations of the primary tumor, including the tumor margin, tumor center, and near adipose tissue. In PDAC, ES-TLS+ tumors are enriched with IgG1-like transformed memory B cells and memory CD4+ T cells with persistent immune memory function and reduced tumor mutational burden (TMB) (95). In addition, the density of CD3+ and CD8+ T cells and CD20+ B cells is significantly higher in ES-TLS tumor tissue. CD8+ T cells infiltrate lymphocyte clusters in and around the marginal area of the follicular center. M-TLSs develop GC, HEVs, and other structures. B cells within GCs are highly proliferative (Ki67+) and express BCL6 (95, 96). B cells exhibit an increased rate of somatic hypermutation. In addition, they act as antigen-presenting cells (APCs) and produce antitumor antibodies, enhancing antitumor humoral immunity (97). The overall level of T-cell infiltration in M-TLS+ tumors is comparable to that in ES-TLS but is more enriched with activated CD4+ T memory cells and naive B cells. Higher CD8+ T-cell densities within the tumor and circulation can influence TLS formation. Similar to CD8+ T cells, FOXP3+ Tregs are significantly enriched in lymphoid aggregates. Although FOXP3+ Tregs are usually present as pro-oncogenic factors, TLS+ tumors have a higher CD8:Treg ratio in the stroma and promote antitumor immunity (98). In addition to FOXP3+ Tregs, marginal tumor regions have more subpopulations with an immunosuppressive phenotype, including CD163+ macrophages, CD11b+ neutrophils, and CD274 (PD-L1)+ tumor cells (98). CD21+ FDCs act as antigen-presenting cells to regulate the recruitment and differentiation of B cells in GCs and regulate the development of T cells through the production of IL-7 (99). Macrophages in TLSs express CD163 at a higher density in the non-TLS stroma than in TLSs and cause a decrease in antigen presentation and T-cell activation in TLSs (100). CD68+ macrophages are distributed in TLS regions in PC. CD163+ and CD68+ macrophages can be found in vaccine-based immunotherapy TLSs (101). In contrast, another study showed that they exist only in regions outside TLSs (96). Mature LAMP+ DCs have a strong antigen-presenting role, and B cells may promote DC maturation (102). Although some studies have shown that NK cells are not found in PDAC tumor tissue TLSs, other studies have found anti-NCR1+ NK cells in non-functional pancreatic neuroendocrine tumors (NF-PanNETs), which further reflects the heterogeneous nature of TLSs (103). One study explored the mechanisms involved in M-TLS+ tumors. Pathways related to reactive oxygen species, MTORC1 signaling, and oxidative phosphorylation were significantly enriched, whereas TGF-β signaling was significantly downregulated. These features correlate with tumor responsiveness to immunotherapy (95).

### 3.4 Patient prognosis

TLSs are rich in TILs and have structural features that allow them direct contact with the tumor, exerting their antitumor immune effects effectively and rapidly. Several studies have found that intratumor TLSs in colorectal cancer (104), gastric cancer (105), and hepatocellular carcinoma (106) tissues contribute to good patient prognosis. There have been some studies on how prognosis relates to the occurrence of TLSs in PC. Tanaka et al. analyzed tumor tissues from 162 PDAC patients and found TLSs in the pathological tissues of 112 patients. TLS formation was associated with high levels of CD4+ TILs, CD8+ TILs, and CD45RO+ TILs but not with FOXP3+ TIL levels. TLS was associated with prolonged survival (p < 0.0001) and good outcomes of adjuvant therapy in PC patients (100). Hiraoka et al. graded TLOs (**Table 3**), classifying patients as having intratumoral TLOs (Grades 3 and 4) or peritumoral only TLOs (Grades 1 and 2). The mean survival of PC patients with intratumoral TLOs was notably greater than that of PC patients without intratumoral TLOs (median 42.67 *vs.* median 15.53 months), and intratumoral TLO presence was an independent predictor for the OS and DFS of patients. In addition, tumor-infiltrating CD4+ and CD8+ T cells were significantly elevated in TLO+ tumor tissues, providing an antitumor immune microenvironment (87). Kuwabara et al. divided 140 patients into a surgery-first (SF) group and a group receiving neoadjuvant chemotherapy (NAC). Immunohistochemical analysis found that the NAC group had a higher proportion of antitumor immune components within TLOs (CD8+ T lymphocytes, HEVs, CD163+ macrophages, etc.) compared to the SF group; in contrast, immunosuppressive cellular components were significantly lower. Patients in the NAC group had a better prognosis. The TLO-to-tumor ratio was shown by multivariate analysis to act as an independent prognostic factor (107). TLOs may also be biological indicators to assess prognosis after radiotherapy. The presence of TLOs in tissues of Grade 1 or 2 (G1/G2) NF-PanNETs also predicts a good prognosis (103). There are few studies on the relationship between peritumor TLSs and prognosis, and the formation of PDAC TLSs in the peritumor stroma may be associated with better prognosis in patients. In conclusion, although there are few clinical studies related to TLSs in PC, TLSs are associated with multiple clinical outcomes (lymph node metastasis, vascular infiltration, and TNM stage). The number of TLSs does not affect patient prognosis.

**Table 3:** The grade of TLOs in PC.

Grade	Main localization of TLO	Frequency
Grade 0	No TLO	0
Grade 1	Peritumoral TLOs	0–0.4%
Grade 2	Peritumoral TLOs	>0.4%
Grade 3	Intratumoral TLOs	0.1–1.0%
Grade 4	Intratumoral TLOs	>1.0%

TLO, tertiary lymphoid organ; PC, pancreatic cancer.

## 4 Potential clinical applications

Because TLS formation is associated with good patient prognosis, it has potential use in antitumor therapy. Some studies have suggested that induction of TLSs may become a new avenue for tumor immunotherapy (93). In colorectal cancer, T-lymphocyte infiltration is induced by injection of DCs expressing the transcription factor T-bet, with TLS formation (108). CCL21 may serve as a target for TLS formation in hepatocellular carcinoma, and its combination with an anti-CD25 monoclonal antibody may improve antitumor efficacy for this cancer (109).

An HEV acts as a channel for delivering TILs to the tumor and is equally capable of acting as a pathway for delivering antitumor drugs. As the monoclonal antibody MECA79 recognizes HEV-expressing peripheral node addressin (PNAd) proteins, some investigators have used MECA79 NP to recognize HEVs in PDAC tissues to deliver the chemotherapeutic drug paclitaxel to tumor tissues, resulting in a significant reduction in tumor size and abnormal intratumor angiogenesis. In the future, using MECA79 NP to identify HEVs can be employed for the delivery of multiple drugs (110). Combination treatment with anti-VEGFR2 and anti-PDL-1 successfully induces HEV formation in the Rip1-Tag2 pancreatic neuroendocrine tumor model (111). HEVs enhance the extent and activity of lymphocyte infiltration by activating the LTβR signaling pathway. Johansson et al. found that LIGHT-VTP normalizes the intratumor vascular system and induces a large number of effector T cells and memory T cells, which enter the tumor through the normal vascular system, thus contributing to the formation of TLSs in the tumor (112). The combination of low-dose cyclophosphamide and allogeneic PDAC vaccine (GVAX) resulted in the depletion of Tregs in the TME, thereby altering its immunosuppressive properties. Of 39 patients treated with this therapy, 33 developed vaccine-induced intratumor TLSs after 2 weeks of vaccine treatment (101). Upregulation of Th17 cells in TLSs with Treg cell inhibition resulted in improved survival. Thus, by inducing the formation of intratumoral TLSs, there is hope that “cold tumors” will be transformed and immunotherapy will be effective (101).

KRAS mutations are present in 90% of PCs, and TILs can target mutant KRAS and inhibit tumor growth. As discussed above, B cells exhibit a dual role in tumor development: a favorable prognostic role associated with promoting TLS formation and a protumor-development role. Tumor-infiltrating B (TIB) cells produce KRAS mutation-specific IgG, and IgG1 monoclonal antibodies are able to interfere with the growth of tumors with KRAS mutations (113). Thus, TIB cells are a potential antitumor antibody molecule targeting neoantigens in the future. Pearce et al. explored immune checkpoint blockade therapy in PDAC patients. TIGIT ligands are widely expressed, with PDL-1 and CD155 expressed within the T-cell compartment of TLSs and colocalized with PD-1+TIGIT+CD8+ T cells in TLSs. CD155 is a TIGIT ligand that inhibits cytokine production, and TIGIT may be a therapeutic target. Combined application of anti-PD-1 and TIGIT blockade therapy promote IFN-γ secretion and T-cell proliferation and inhibit tumor progression (114). Thus, dual immune checkpoint blockade may become a new avenue of immunotherapy for PC. Oncolytic adenovirus (TILT-123) produces immunostimulatory effects on T cells, and TILs are able to deliver TILT-123 to tumor tissues to exert antitumor effects (115).

## 5 Factors influencing formation of TLSs

TILs in the microenvironment play an indispensable role in TLS formation, and they are able to regulate the development of disease by secreting chemicals. They are also influenced by a variety of factors and pathways.

Multiple components of the TME and signaling pathways are capable of influencing TILs. USP22 in PC cells interacts with SAGA/STAGA to affect the immune microenvironment, and USP22 deficiency promotes T-cell and NK cell infiltration and inhibits tumor metastasis (116). Lipid metabolism can also affect TILs, and forced expression of ACADVL can metabolically reprogram tumor-specific T cells and improve the survival and persistence of intratumor T cells in a PDAC-engineered mouse model (117). Knockdown of PAK1 increases intratumor CD4+ and CD8+ TILs and eliminates the protective effect of pancreatic stellate cells (PSCs) on tumor cells, allowing them to be killed by the immune system (118). Overactivation of cholinergic signaling in PC can promote tumor growth by suppressing intratumor T-cell responses (119). The role of various chemokines and receptors in regulating TILs cannot be ignored. Blockade of CXCR4 promotes T-cell infiltration in tumors, reduces circulating Treg cells, and acts synergistically with anti-PD-1 to exert immune-promoting effects (120). Multiple chemokines are significantly overexpressed in PC tissues (CXCL5, CXCL9, and CXCL10). CXCL9 reduces CD8+ TILs in the TME, and STAT3 inhibition is able to restore proliferation, activation, and antitumor cytokine secretion of CXCL9-suppressed CD8+ TILs (121). Upregulation of CCL5 enables tumor FOXP3+ Treg cell aggregation in PDAC (122). Neoadjuvant or immunotherapy can also have an effect on TILs. Increased infiltration of CD3+ T cells is observed in CRT-treated tumors, including CD3+CD8+ T cells and CD3+CD4+FOXP3+ Treg cells but with no difference in CD68+ macrophages (123). Abraxane in combination with hIL15-ABD causes CD8+ T cells to secrete increased IFN-γ and suppresses the production of immunosuppressive markers (FOXP3, VEGF, etc.) (124). The combination of CCKR blockade results in a significant decrease in tumor FOXP3+ Tregs and an increase in CD4+ and CD8+ lymphocytes (125). Tumor tissues of PC patients who undergo neoadjuvant therapy have elevated CD4+ and CD8+ TILs and a lower postoperative recurrence rate.

Estrogen signaling plays a role in a variety of tumors (126); therefore, some investigators have explored the potential role of estrogen signaling in TLS formation in PC. Estrogen receptors are expressed in PC tissues, such as ERα, ERβ, and GPER, all of which are associated with reduced tumor malignancy and good prognosis. Among them, ERα+ and ERβ+ expression is upregulated in tumor cells. In addition, the positivity of all these ERs can affect the TME and promote active immune cells as well as the formation of intratumoral TLSs (127). Nerve fiber density (NFD) is the number of small nerve fibers invaded by tumor cells. According to one study, high NFD in the TME is linked to increased survival in PDAC patients. Lymphoid aggregates surround these small nerve fibers. Patients with five or more lymphoid aggregates and a high NFD have a better prognosis (128). The presence of TLSs is associated with higher levels of IL-2 in the stroma and lower levels of IL-2 in the tumor compartment. Low circulating IL-2 levels are associated with the formation of local TLSs, and therefore, serum IL-2 levels may serve as a marker for TLS prediction (129). In contrast to chemotherapy, which enables the production and development of TLOs in tumor tissue, stereotactic body radiotherapy (SBRT) can induce immunogenic tumor cell death, initiate cytotoxic T cells, increase the ratio of PD-1+ T cells, and decrease TLSs in PC. Its effect on TLSs can revert after the termination of SBRT treatment (130, 131) (**Table 4**).

**Table 4:** The factors regulating TILs and TLSs.

Regulatory Factors	Target cell/tissue	Regulation results	Reference
USP22/SAGA/STAGA	T cells and NK cells	Inhibit tumor metastasis	(116)
ACADVL	Tumor-specific T cells	Improve survival and persistence of intratumor T cells	(117)
PAK1 knockdown	CD4+ and CD8+ TILs	Eliminate the protective effect of PSCs on tumor cells	(118)
CXCR4 blockade	T cells	Promote T-cell infiltration Reduce circulating Treg cells	(120)
Cholinergic signaling	T cells	Suppress the intratumor T-cell response	(119)
CXCL9	CD8+ TIL	Reduce CD8+ TILs in the TME	(121)
CCL5	Treg cells	Treg cell aggregation	(122)
CRT-treated	CD3+/CD8+ T and Treg cells	Increase T-cell infiltration	(123)
Abraxane in combination with hIL15-ABD	CD8+ T cells	Increase IFN-γ and suppress production of immunosuppressive markers	(124)
CCKR blockage	CD4+/CD8+ T and Treg cells	Decrease FOXP3+ Tregs Increase CD4+ and CD8+ TILs	(125)
ERα+ and ERβ+	TLS	Promote immune cell activation Intratumoral TLS formation	(127)
Low circulating IL-2	TLS	TLS formation	(129)
SBRT	T cells and TLS	Induce tumor cell death Increase the ratio of PD-1+ T cells Decrease TLSs	(130)

TILs, tumor-infiltrating lymphocytes; TLS, tertiary lymphoid structure; SBRT, stereotactic body radiotherapy.

## 6 Summary and discussion

TLS formation, as an important link in the antitumor immune response, may alter the response. TLSs are present in and around PC, and PC patients have a better prognosis when intratumoral TLSs are present. In addition to being a prognostic marker, it is possible to assess the effect of different treatment modalities by detecting the formation or reduction of TLSs. Induction of TLS formation may become a new target for immunotherapy and provide new ideas for the treatment of “cold tumors”. In addition to TLS formation in PC, TLS formation is also a hallmark of pancreatic-associated immune disease autoimmune pancreatitis (AIP), which is currently considered to be T cell-mediated chronic pancreatitis, and research on the long-term treatment and management of patients with AIP is currently limited and needs to be expanded (132). Some studies have found TLS formation in the pancreatic tissue of AIP patients, in addition to overexpression of LTα, β, and LTβR target genes and elevated expression of CXCL13, CCL19, CCL21, CCL1, and B-cell activating factors in serum samples (133, 134). P21 is involved in inflammatory effects in AIP. Its deficiency prevents early pancreatic injury and reduces inflammation in AIP. Suppressed activation of the NF-κB pathway was observed in pancreatic follicular cells. P21+ and P21− mice have similar TLOs, autoantibody levels, and elevated IgG levels (135). In addition to the conventional application of corticosteroids, some investigators have treated AIP by targeted inhibition of the LTβR pathway. Application of LTβR-Ig and anti-CD20 treatment results in significant improvement in AIP, and blockade of LTβR signaling leads to a decrease in tissues with TLSs (136). In 1996, an investigator found that in rat insulin promoter-LT (RIP-LT)-transgenic mice, chronic inflammatory lesions in the kidney and pancreas were similar to lymph nodes in terms of cellular composition, T- and B-cell areas, and morphological features of HEVs (68). Similar lymph-like structures could be found in transgenic mice expressing BLC in pancreatic islets. These features are dependent on B lymphocytes and LTα1β2, and blocking LTα1β2 reverses this phenomenon (137). TLS promotes autoimmunity as well as chronic inflammation in autoimmune diabetes. In early diabetic mice, progression from peri-insulitis to endo-insulitis is characterized by local upregulation of LTα/β, CXCL13, and CCL19 and infiltration of follicular B cells into lymphoid aggregates with T/B-cell partitioning, the FDC network, and GC B-cell differentiation. In contrast, the incidence of TLS in pancreatic tissues of advanced diabetic mice is significantly reduced (138). Silke et al. reported a case of large lymphocyte infiltration similar to TLSs found in the islet tissue of a patient with chronic type 1 diabetes (T1D) (139).

Although TLSs have advantages, their practical application in the clinical setting still faces some challenges. The number of TILs within TLSs is much higher than the number of TILs outside TLSs. There is a lack of criteria that clearly distinguish TLSs from TILs, and more research is needed to define TLSs, including the minimum area, the minimum number of lymphocytes in TLS, and the minimum value of density. As research on TLSs in PC remains limited, more large-scale multicenter clinical trials are required to investigate in depth the relationship between TLSs in different locations (intratumor and peritumor) and different states (early-stage and mature) and patient prognosis. Further exploration of whether peritumor TLSs can be converted to intratumor TLSs is also needed. At present, the detection of TLSs can only be performed at the tissue level, and the lack of standardized detection tools and subjectivity of TLS determination by different pathologists may lead to somewhat different detection. In addition to invasive tests, the cost and time required for patients to be examined are also problems. Therefore, in the future, there is a need to develop cheaper and faster markers for the non-invasive detection of TLSs through human body fluids (serum, saliva, urine, etc.). Finally, because of the heterogeneous and dynamic nature of both the human TME and TLSs, the findings obtained from *in vivo* experiments in mouse models do not represent their applicability in patients with PC. Therefore, scientists need to conduct more *in vitro* and *in vivo* experiments to fully explore the mechanisms.

In conclusion, the emergence of TLSs brings some hope for PC patients in the current situation in which immunotherapy for PC is facing certain dilemmas. TLS antitumor effects have been demonstrated in many tumors. Inducing the production of TLSs may become a new avenue for individualized immunotherapy for PC patients.

## Author contributions

XLZ and CG contributed to the idea for this review article. The literature search and analysis were performed by XLZ, CG, JG, and WS. Further collection, analysis, and arrangement were accomplished by XLZ and CG. The first draft of the manuscript was written by XLZ. YC and XYZ helped with the final revision of this manuscript. All authors reviewed and approved the final manuscript.
